# The Effect of Supplementing Mushroom Growing Substrates on the Bioactive Compounds, Antimicrobial Activity, and Antioxidant Activity of *Pleurotus ostreatus*

**DOI:** 10.1155/2022/9436614

**Published:** 2022-06-27

**Authors:** Senzosenkosi Surprise Mkhize, Mthokozisi Blessing Cedric Simelane, Ishmael Nkoana Mongalo, Ofentse Jacob Pooe

**Affiliations:** ^1^Discipline of Biochemistry, Westville Campus, University of KwaZulu-Natal, Private Bag X54001, Durban 4000, South Africa; ^2^Department of Biochemistry, University of Johannesburg, P.O. Box 524, Auckland Park 2006, South Africa; ^3^College of Agriculture and Environmental Science (CAES) Laboratories, University of South Africa, Private BagX06, Johannesburg 0710, South Africa

## Abstract

*Pleurotus ostreatus* mushroom contains important bioactive compounds and has several biological activities; however, mushroom growing substrates have major influence on chemical and functional characteristics of the mushroom. Hence, the study aimed to evaluate the influence of supplementing mushroom growing substrates with wheat bran (WB) towards yield/productivity, bioactive compounds, and antimicrobial and antioxidant activity of *P*. *ostreatus*. The mushroom was cultivated on sugarcane substrates supplemented with increasing levels of WB (0%–20%). The mushroom extracts were screened for bioactive compounds using gas chromatography-mass spectrometry (GC-MS). Antimicrobial activity was carried out using microplate assay, while antioxidant potential was investigated using reducing power assay. The addition of supplements on mushroom growing substrates had an influence on mushroom yield; hence, higher supplementation (18% and 20%) produced higher yield. The GC-MS revealed several bioactive compounds with known activity, such as vitamin E, phenol, fatty acids, and terpenoids. Concentration-dependent antioxidant activity was observed; hence, extracts at higher concentrations gave significantly higher reducing power. The *P. ostreatus* extract had antimicrobial activity against all the tested organisms, with *S*. *aureus* showing high susceptibility to most of the extracts. However, mushrooms grown on bagasse substrates supplemented with 14% (0.02 mg/ml) and 20% WB (0.08 mg/ml) proved to have better antimicrobial activity on *Escherichia coli*. The difference in susceptibility demonstrates that substrates type and composition could have an influence on bioactive compounds found within mushrooms, also influencing medicinal properties of edible mushroom. Thus, supplementing mushroom growing substrates not only improve yield, but also can contribute to bioactive compounds with medicinal potential.

## 1. Introduction

Mushrooms are usually defined as the macrofungi with fruiting body which could be either above (hypogenous) or underground (epigeous) and could be seen with naked eyes; hence, it can be picked [1, 2]. The mushrooms are being gradually recognized as important food due to their contribution towards human health, disease, and nutrition [2]. Hence, they are being utilized throughout the world as food, drugs, and tonics [3]. Among all the cultivated mushrooms, oyster mushroom species are one of the most cultivated mushrooms worldwide following *Agaricus bisporus* [4]. This is due to the fact that *Pleurotus* spp. are easily cultivated at low production cost with high yield and biological efficiency [5]. Furthermore, the *Pleurotus* spp. could be easily cultivated on a number of readily available substrates [6].

These saprophytic fungi have been reported to contain numerous metabolites, which exhibit many important pharmacological activities [7]; thus, oyster mushrooms have been recognized to be highly medicinal due to their content of bioactive metabolites, which could potentially be used to produce a variety of important pharmaceutical products [8]. The bioactive metabolites such as phenolic compounds, polyketides, terpenes, and steroids, which are usually found in *Pleurotus* mushrooms, have previously been reported to be medically active in several therapies [4]. For example, compounds such as pentadecane and Phenol, 2,4-bis (1,1-dimethylethyl), which could be found in *P. ostreatus* mushrooms have both antioxidant and antimicrobial properties [9, 10]. Furthermore, it is noteworthy that natural antioxidants such as phenolic compounds are in high demand due to their potential in the treatment of several diseases such as diabetes and cardiovascular disorders and anticancer, anti-inflammatory, and antimicrobial activities, besides their potential applications in the food and pharmaceutical sectors [11–14].

It is important to also note that the type or characteristics of the growing substrates significantly influence the content of certain bioactive compounds within the mushrooms [15, 16]. Therefore, it is important to select the good substrates, which could increase the content and variations of metabolites such as phenolics in mushrooms [17]. Furthermore, mushroom growing substrates have previously been supplemented with different supplements in order to promote rapid growth and productivity of mushrooms [18, 19]. Supplements such as wheat bran have been used as a source of carbohydrates and nitrogen to the main substrates [20], since most of the substrates do not have enough nitrogen required by mushroom [21]. Hence, it has been established that wheat bran supplement improves yield/productivity of *P. ostreatus* mushrooms [18]. It was therefore the aim of the study to evaluate the influence of wheat bran supplement towards mushroom productivity, content of bioactive compounds, and biological activity of oyster mushroom. Thus, information scarcity on utilizing wheat bran supplements to improve quality, content of bioactive compounds, productivity, antimicrobial activity, and antioxidant property of *Pleurotus ostreatus* mushrooms would be fixed. Hence, an alternative source of antibiotic towards the resurgence of multidrug resistance and nosocomial infections could potentially be established from such mushrooms. Therefore, the aim of the current work is to compare the antimicrobial and antioxidant activity of mushrooms cultivated at different quantities of sugar cane bagasse and sugar cane tops and, furthermore, to identify and quantify possible compounds which could well explain biological activity of such extracts using gas chromatography-mass spectrometry.

## 2. Materials and Methods

### 2.1. *P*. *ostreatus* Mushroom Cultivation Procedure

*P*. *ostreatus* mushrooms were cultivated on locally available substrate/waste materials namely sugar cane bagasse and sugar cane tops. Few modifications from the methods by [18] were incorporated in the process of mushroom cultivation. The four-step process was followed for the cultivation of *P*. *ostreatus*: (a) preculturing of *P*. *ostreatus* on PDA, (b) spawn preparation, (c) substrate preparation and inoculation, and (d) *P*. *ostreatus* fruiting. The test *P*. *ostreatus* mushroom was obtained from Cedara College of Agriculture at Pietermaritzburg in Kwa-Zulu Natal (South Africa), where it was previously identified and characterised.

The *P. ostreatus* mushroom strain was initially precultured on potato dextrose agar (PDA) and thereafter incubated under dark environment at ±25°C till mycelia fully covered the PDA plate. The precultured *P*. *ostreatus* strain was then stored at 4°C, hence maintained as mother culture for further processing. *P*. *ostreatus* mushroom spawn was prepared following a modified method outlined by [22] using birds seed grains that was soaked in overnight in distilled. Briefly 4 g of the soaked birds seed grains were mixed with 1g gypsum (CaSO. 4·2H_2_O) and 300 g calcium carbonate (CaCO3) and autoclaved. The grains were then inoculated aseptically with the previously grown mushroom cultures and thereafter incubated under dark environment at ±25°C, until mycelia fully colonized the bird seed grains. The prepared mushroom spawn was also stored at 4°C for further processing. The locally available substrates (sugarcane bagasse and sugar cane tops) were sparingly sprinkled with H_2_O till 65% moisture was achieved. Thereafter, the substrates were supplemented with wheat bran at levels of 0%, 2%, 18%, and 20% wheat bran (WB), respectively. These levels of supplements were thoroughly mixed with the substrates and then pasteurised at 60–65°C for six hours and allowed to cool at room temperature [23]. The pasteurised substrates were then inoculated with the previously prepared spawn, which was later incubated under dark till mycelia fully colonised the substrates. Once the substrates were fully colonized by mushroom mycelia, they were removed from dark environment into the fruiting room that was made of 30% shade cloth. The *P*. *ostreatus* mushrooms fruited under ambient temperatures at constant fogging to achieve 60% moisture which is ideal for fruiting of oyster mushrooms.

### 2.2. Carbon to Nitrogen Ratio (C/N) of Supplemented Substrates and Mushroom Yield/Productivity

The supplemented substrates (sugar cane tops and sugar cane bagasse) were analysed for total carbon and nitrogen composition following a modified method by [24]. The C and N within substrates were analysed using CHN analyser (Leco, Moenchengladbach, Germany) following the combustion method, whereby 3 mg of dried substrates were analysed in triplicate. Thereafter, the C/N ratio was calculated from the mean of the results obtained and used to confirm the degree of condensation of organic compounds. The yield of the produced mushrooms was calculated according to method extrapolated from [18]; hence, the following equation was used:

(1) $$\begin{array}{c} \text{MY}=\left[\frac{\text{Weight of fresh mushroom harvested}\left(g\right)}{\text{fresh substrate weight}}\right]. \end{array}$$

### 2.3. Preparation of Mushroom Extracts

A slightly modified method by Chowdhury et al. [25] was used for the extraction and preparation of mushroom extract. The freshly harvested mushrooms were sundried under transparent tunnel with 30% shade cloth. Then the dried mushrooms were milled into powder using a Scientec hammer mill, resulting into a 2 mm mesh powder. Thereafter, 100 g of powdered mushroom was dissolved into 250 ml methanol and incubated in shaker set at 200 rpm for 24 h at 25°C. The mushroom extract was filtered using Whatman No. 1 filter paper and evaporated to semidryness using fume mode. The semidry *P*. *ostreatus* mushroom extract was then stored at 4°C for further analysis.

### 2.4. Screening of Bioactive Compounds Using Gas Chromatography-Mass Spectrometry (GCMS)

Well established method by Daffodil et al. [26] was used to screen for the bioactive compounds within the mushrooms. For GCMS analysis, 3 mg of extract was mixed with 10% methanol and 90% dichloromethane (DCM) and shaken to dissolve into a homogenous mixture. The GC-MS analysis of the extract was conducted using the Shimadzu GC-MS solutions system, whereby the gas chromatography interfaced with the mass spectrometer (GC-MS) which had Elitel and a fused silica capillary column (30 mm × 0.25 mm 1D X1 *μ*Mdf, consist of 100% Dimethyl poly siloxane). Electron ionization system with ionizing energy of 70 eV was used for the GCMS detection. The carrier gas named Helium (99.999%) was used at the constant flowrate of 1 ml/min, with the injection volume of 8.00 *μ*l, and split ratio of 10 : 1.250°C was the injector temperature and 28°C being the ion source temperature. The oven was programmed at 110°C (2 min isothermal) and was increasing at 10°C/min to 200°C and 5°C/min to 280°C, then ended with 9 min isothermal which was at 280°C. The 70 eV was used for the mass spectra with scanning interval of 0.5 seconds, having fragments from 45 to 450 Da. The GC had the running time of 36 min in total at which the relative amount in percentage of each component was calculated using comparison of the average peak area with the total areas. The GC-MS mass spectrum was interpreted using database of National Institute Standard Technology (NIST), which has more than 62000 patterns in their library.

### 2.5. Antimicrobial Activity Assay

The minimum inhibitory concentration (MIC) of mushroom extracts was determined for *Escherichia Coli*, *Staphylococcus aureus*, *Candida albicans,* and *Cryptococcus neoformans* using the microplate dilution assay [27]. About 100 *μ*l of nutrient broth was added to all the 96 well microtitter plates. About 100 *μ*l of mushroom extract (5 mg/ml) dissolved in 1% DMSO was thereafter serially diluted throughout the rows in the 96 well microtitter plates. About 100 *μ*l bacterial culture set at 0.5 McFarland standard was then added to all the wells of the 96 well microtitter plates which were incubated at 37°C for 24 hours. The *p*-Iodo-nitrotetrazolium violet (INT) solution (40 *μ*l of 0.2 mg/ml) was thereafter added into 96 well microtitter plates and incubated at 37°C for 30 min. The red colour indicated the growth of the microorganism, fermenting INT into formazan. MIC was recorded as the lowest concentration of mushroom extract that completely inhibited the microbial growth of organisms.

### 2.6. Reducing Power Assay

The *P*. *ostreatus* ability to reduce free radicals was evaluated using modified method reported by Ayeni et al. [28]. About 2.5 mL of the methanolic mushroom extracts at various concentrations (10–800 *μ*g/ml) were mixed with 0.2 M phosphate buffer (pH 6.6) together with 1% potassium ferricyanide. The mixture was incubated at 50°C for 20 min, and thereafter, 10% trichloroacetic was added, and the solution was centrifuged for 10 min at 1000 rpm. Immediately after centrifugation, 2.5 ml of the upper layer of the solution was taken out and mixed with equal ratio of distilled water and FeCl_3_ (0.5 mL, 0.1%). Absorbance was measured at 700 nm (96 well plate reader); hence, higher absorbance indicated better reducing power of mushroom extracts.

### 2.7. Statistical Analysis of Results

All experiments were repeated independently, in triplicate. Data generated were calculated using SPSS original 6.0 and Graph Pad Prism. The results are reported as mean ± S.E.M. The statistical differences were determined using one-way analysis of variance (ANOVA), followed by Tukey-Kramer multiple comparison test. The values were considered statistically significant where *p* ≤ 0.05.

## 3. Results and Discussion

### 3.1. The C/N Ratio of Growing Substrates Together with P. ostreatus Yield

**Figure 1:**
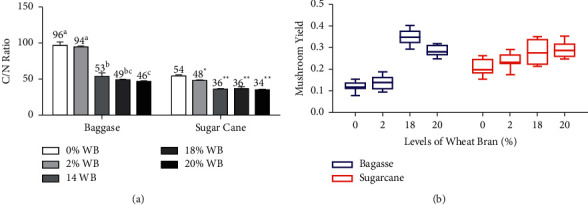
(a) The C/N ratio of mushroom growing substrates, which was supplemented with increasing levels of WB and (b) the yield of *P*. *ostreatus* mushroom, which was grown on various supplemented substrates. C/N: carbon to nitrogen; WB: wheat bran.

The *Pleurotus* mushrooms have the greatest advantage of being cultivated in a simple way within tropical climates. Such mushrooms can be grown on both lignin and cellulose rich substrates which are readily obtainable [29]. Even though the *Pleurotus* mushrooms have such advantages, it should be emphasised that the growth and yield performance of *P*. *otreatus* are dependent on the carbon to nitrogen ratio (C/N) of the growing substrates [30]. Hence, previous studies have reported that substrates should be supplemented with nitrogen and carbon source to obtain optimum C/N for the growth of mushroom [31]. Therefore, mushroom growing substrates should be formulated in such a way that they contains balanced C/N ratio [30]. In our study, two different substrates (sugar cane tops and sugar cane bagasse) were supplemented with varying levels of wheat bran (0%, 2%, 14%, 18%, and 20%) in order to obtain an optimum C/N ratio which would improve the growth and yield performance of *P. otreatus* mushrooms. From the results on Figure 1(a), it is noted that the unsupplemented substrates (0%) and lower levels of WB supplemented substrates (2%) had significantly higher C/N, when compared to higher levels of supplementations (14%, 18% and 20%). This was especially observed for bagasse supplemented with 0% and 2% WB, which had significantly (*p* < 0.05) higher C/N ratio (96 : 1 and 94 : 1), when compared to bagasse supplemented with 14%, 18%, and 20% WB. Similar trend was also observed for the sugar cane supplemented substrates, with the exception being the fact that sugar cane substrates had lower C/N ratio when compared to bagasse substrates. The obtained C/N ratio for higher levels of supplementation for bagasse substrates was in line with an ideal C/N value reported by Duprat [32] who stated that an ideal C/N value for *P*. *ostreatus* grown agro-industrial waste should range from 25 to 50 : 1. The sugar cane substrates had an ideal range for C/N ratio regardless of the effect of wheat bran supplementation. Our study confirms that as C/N ratio decreases, the yield could potentially increase; hence, it was noted that higher levels of supplementation that had lower C/N ration and hence produced better yield (Figure 1(b)), when compared to the lower levels of supplementation which had higher C/N ratio. Therefore, increasing the levels of wheat bran supplement in mushroom growing substrates might have probably resulted in balanced or optimum C/N, hence influencing the yield performance of *Pleurotus* spp. mushroom. Our findings were in line with the results reported by [33] who stated that substrates with lower C/N ratios result in higher mushroom yield. The C/N ratios that were obtained, were influenced by the level of wheat bran supplemented into the substrates; hence, it was observed that for both substrates, an increase in wheat bran level resulted in the increase in mushroom yield (Figure 1(b)). Such findings corroborates with the findings of [34–36], who reported that an increase supplementation level within the base substrates result in increased mushroom yield, which is of great interest to mushroom farmers.

### 3.2. The Antioxidant Property of *P*. *ostreatus* Mushroom

**Figure 2:**
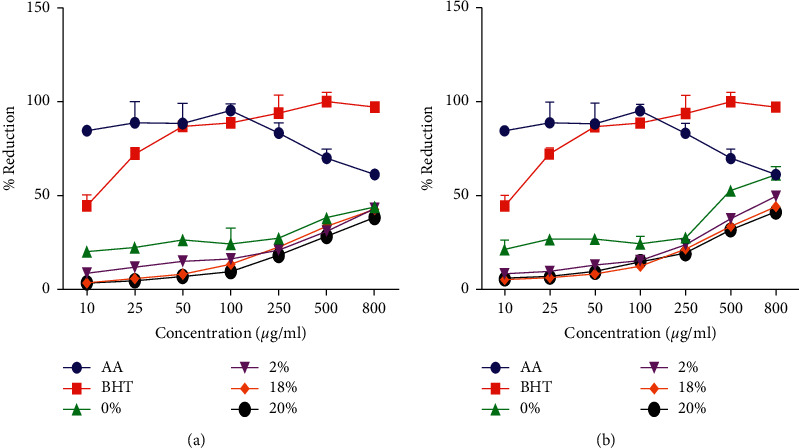
The Reducing power of *P*. *ostreatus* mushroom grown on various supplemented substrates: (a) sugarcane bagasse base substrates supplemented with different levels of wheat bran); (b) sugar cane tops base substrates supplemented with different levels of wheat bran).

Mushrooms have been reported to have protective role just like many plants, they potentially protect organisms against oxidative stress; hence, they produce phytocompounds with antioxidant activity, which prevent formation of free radicals, which forms diseases due to the reactive oxygen species (ROS) and reactive nitrogen species (RNS) [37]. Hence, previous authors have stated that the consumption of Oyster mushrooms may protect the human body since Oyster mushrooms have radical scavenging activity [38]. The present study revealed that methanolic extract of *P*. *ostreatus* mushroom grown on both unsupplemented and wheat bran supplemented substrates (sugar cane tops and sugar cane bagasse) have antioxidant property. Figures 2(a) and 2(b) indicate that *P*. *ostreatus* mushrooms have reducing power, which was concentration-dependent; hence, the reducing power was observed to be increasing as the concentration increased throughout. Such trend of concentration dependency of reducing power of *P*. *ostreatus* mushroom is in line with the results obtained by [39], who also demonstrated that the reducing power of methanol extract from five ear mushrooms was dose-dependent. Thus, the observed reducing power (ability) of *P*. *ostreatus* mushroom might be due to the fact that mushrooms have the hydrogen donating ability which breaks the free radical chain [40]. When comparing the reducing power of different supplement levels, it was observed from Figures 2(a) and 2(b) that unsupplemented (0%) substrates had higher reducing power followed by lower supplementation (2% WB) compared to the rest of supplemented substrates. The highest reducing power was observed at the concentration of 800 *μ*g/ml, with a reducing power of 42% observed on unsupplemented (0%) sugar cane bagasse. The unsupplemented (0%) sugar cane tops were also observed to have higher reducing power of 61% with a concentration of 800 *μ*g/ml, which was similar to the reducing power of the control namely ascorbic acid (A. A), which also had a reducing power of 61% at 800 *μ*g/ml. Such variations in reducing power of differently supplemented substrates was probably due to the differences in the amount of reductones such as phenolics and flavonoids, which have antioxidant ability through breaking free radical chain by donating a hydrogen atom [41].

### 3.3. Antimicrobial Activity of *P*. *ostreatus* Mushroom Extracts

Besides the antioxidant property (reducing power) of mushrooms, the current study also confirmed that the *P*. *ostreatus* mushroom also have the antibacterial and antifungal activity as observed on Tables 1 and 2. The results in Tables 1 and 2 reveal that *P*. *ostreatus* grown on wheat bran supplemented substrates have the antimicrobial potential. The methanolic extract of *P*. *ostreatus* mushroom grown on differently supplemented substrates inhibited *Staphylococcus aureus*, *Escherichia coli*, *Candida albicans,* and *Cryptococcus neoformans* with MIC values ranging from 2.5 mg/ml to 0.08 mg/ml. The results in Table 1 indicate that mushroom extracts grown on sugarcane tops had moderate antibacterial activity for *S. aureus* (MIC range from 0.31–0.16 mg/ml) in all levels of WB supplementation, and 20% WB showed moderate antibacterial activity towards *C*. *albicans* (0.31 mg/ml). However, mushroom extracts grown from sugarcane bagasse had good antibacterial activity for supplements such as 14% (0.02 mg/ml) and 20% WB (0.08 mg/ml) for *E*. *coli* bacteria. Furthermore, moderate antibacterial activity was also noted for sugar cane bagasse on bacteria such as *S. aureus* (0.31–0.63 mg/ml), *C. albicans* (0.31–0.63 mg/ml), and *C*. *neoformans* (0.16, 0.31, and 0.63 mg/ml). These results indicate that *P. ostreatus* extract had better activity; hence, the auth [27] have reported that the lower the MIC, the better the activity, and other researchers have stipulated and classified the antimicrobial activity of plant extract as good (MIC < 0.1 mg/mL), moderate (0.1 ≤ MIC ≤ 0.625 mg/mL), and weak (MIC > 0.625 mg/mL) [42]. Thus, such findings support the reports of previous studies which stipulated that *Pleurotus ostreatus* mushrooms have antibacterial and antifungal properties [43].

In general, it was noted that supplementation of substrates with wheat bran had some impact on the antimicrobial activity (antibacterial and antifungal) of *P*. *ostreatus* as observed that the mushrooms grown on unsupplemented substrates (0%) had MIC values which were different to other supplemented substrates; however, other mushrooms grown on supplemented substrates had MIC values which were similar to the MIC values of unsupplemented substrates. For example, it is observed in Table 2 that *Staphylococcus areus* was equally inhibited by *P*. *ostreatus* extract grown on unsupplemented (0%) and supplemented (2% and 18% WB) bagasse substrates since the MIC value of 0.63 mg/ml was noted; however, the only exception was only observed on 14% WB (MIC of 0.16 mg/ml) and 20% WB (MIC of 0.31 mg/ml). Such similarities and differences in terms of inhibitory activities of *P*. *ostreatus* extract were probably due to their content in total phenols and flavonoids as Barros et al. [44] had previously stipulated that the antimicrobial activity of different mushrooms was directly correlated with their content of total phenols and flavonoids.

### 3.4. GCMS Analysis of Compounds within *P*. *ostreatus*

The GC-MS results in Tables 3 and 4 also testify that the mushrooms grown on various supplemented substrates have varying and some have similar compounds which might have played a role in both antimicrobial activity and antioxidant activity, which was observed in our study. Therefore, it could be stipulated that the high antibacterial potency observed for mushrooms grown in bagasse supplemented with 14% WB (MIC of 0.02 mg/ml) and 18% WB (MIC of 0.08 mg/ml) could be due to the presence of compounds such as Pentadecane, Hexadecane, Heptadecane, and Phenol, 2,2′-methylenebis [6-(1,1-dimethylethyl), which are known to have antibacterial activity. Furthermore, the GC-MS analysis have detected several compounds from *Pleurotus ostreatus* mushroom grown on different substrates (sugar cane tops and sugar cane bagasse) with varying levels of wheat bran supplements. Some of these compounds have the known biological activities; however, others do not have known biological activities. The results in Tables 3 and 4 indicate compounds of known activities; hence, compounds which did not have known activities were not included within the Tables.

**Figure 3:**
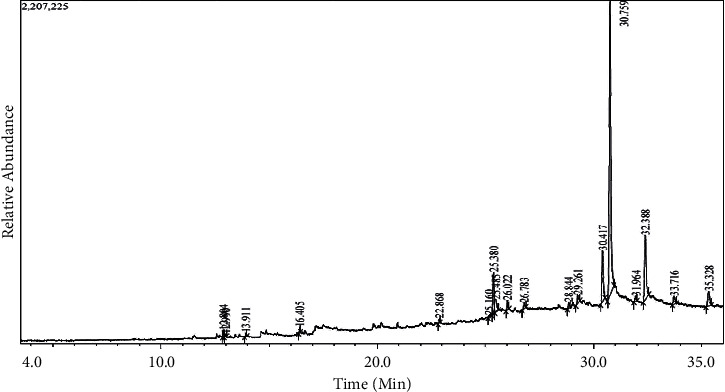
GC-MS chromatogram of methanolic extract of *P*. *ostreatus* mushroom cultivated from sugar cane substrates supplemented with various levels of wheat bran.

**Figure 4:**
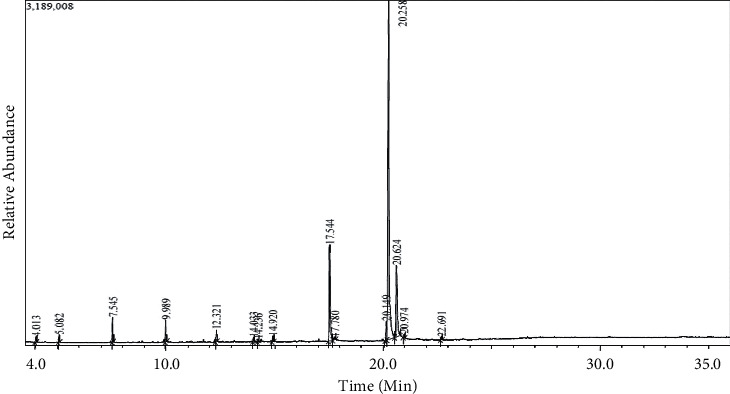
GC-MS chromatogram of methanolic extract of *P*. *ostreatus* mushroom cultivated from sugarcane bagasse supplemented with various levels of wheat bran.

However, the GCMS screening on Tables 3 and 4 indicates that *Pleurotus ostreatus* grown on different substrates with various levels of wheat bran supplementation produced many compounds of which some are similar. The mushrooms grown on sugar cane (Table 3) supplemented with 0%, 18%, and 20% WB had similar compounds such as vitamin E compounds namely beta Tocopherol, gamma Tocopherol, and Alpha-Tocopherol, which are known to have antioxidant property [45]. Furthermore, mushrooms grown on sugarcane bagasse (Table 4) supplemented with 0%, 2%, and 20% WB also had vitamin E compounds. Besides the vitamin E compounds, there were plenty of other bioactive compounds detected by GCMS analyzer, including phenols, terpernoids, fatty acids, and fatty acids derivates as profile chromatogram on Figures 3 and 4 have highlighted some of these compounds found on methanolic extract of *P*. *ostreatus* mushroom. Compounds such as heptadecane, hexadecane, pentadecane, dibutyl phthalate, cholest-22-ene-21-ol, 3,5-dehydro-6-methoxy- and ß-sitosterol which were detected on some of the *Pleurotus ostreatus* mushrooms are well known to have antimicrobial activity [46–51]. Such findings prove that some of the activities observed such antioxidant and antimicrobial were mainly due to the presence of the above-mentioned compounds. However, in vivo studies need to be explored to validate such activities.

## 4. Conclusions

Based on the findings of the study, it can be concluded that *P*. *ostreatus* mushroom produces better yield under a lower C/N ratio, which is influenced by the levels of supplement added into mushroom growing substrates. Furthermore, wheat bran supplement has some influence on the content of bioactive compounds within the *P*. *ostreatus* mushroom; hence, higher levels of supplementation caused the decrease in antioxidant potential (Ferric reducing power) of *P*. *ostreatus*. Therefore, this means the addition of supplements in mushroom growing substrates has the advantage of better yield but with decreased antioxidant property; however, little or no supplements has advantage of higher antioxidant potential but reduced yield. Further research needs to be conducted to confirm the correlation between the content of mushroom compounds towards different substrates.

## Figures and Tables

**Table 1:** Minimum inhibitory concentration (MIC) (mg/ml) of methanol extract of *P*. *ostreatus* mushroom grown on sugar cane tops supplemented with varying levels of wheat bran.

Supplement (WB)	Test organisms			
	*E. coli*	*S. aureus*	*C. albicans*	*C. neoformans*
0%	2.50	0.31	1.25	2.5
2%	2.5	0.16	1.25	1.25
14%	2.5	0.31	2.5	1.25
18%	2.5	0.31	2.5	1.25
20%	1.25	0.16	0.31	1.25

Control drugs				
Vancomycin	0.002	0.001	—	—
Streptomycin	0.025	0.013	—	—
Amphotericin	—	—	0.012	0.004

**Table 2:** Minimum Inhibitory concentration (MIC) (mg/ml) of methanol extract of *P*—*ostreatus* mushroom grown on bagasse substrates supplemented with varying levels of wheat bran.

Supplement (WB)	Test organisms			
	*E. coli*	*S. aureus*	*C. albicans*	*C. neoformans*
0%	2.50	0.63	2.5	1.25
2%	1.25	0.63	0.31	0.63
14%	0.02	0.16	0.31	0.16
18%	2.5	0.63	0.31	0.31
20%	0.08	0.31	0.63	2.5

Control drugs				
Vancomycin	0.002	0.001	—	—
Streptomycin	0.025	0.013	—	—
Amphotericin	—	—	0.012	0.004

**Table 3:** GCMS profiling of methanolic extracts of *P*. *ostreatus* mushroom grown from sugarcane substrates supplemented with varying levels of wheat bran.

Supplement (%)	Peak	RT (min)	Compound	Molecular formula	Area %	Height %	Mol wt
0	1 2 5 6 9 11 13 14 16	12.894 12.990 22.868 25.160 26.022 28.844 30.417 30.759 32.388	Phytol acetate 2-Pentadecanone, 6,10,14-trimethyl- Pentadecanal- cis-11,14-Eicosadienoic acid, tert-butyldimethylsilyl 2-Methyl-Z,Z-3,13-octadecadienol 2H-1-Benzopyran-6-ol, 3,4-dihydro-2,8-dimethyl-beta.Tocopherol.gamma.-Tocopherol Vitamin E	C22H42O2 C18H36O C15H30O C26H50O2Si C19H36O C27H46O2 C28H48O2 C28H48O2 C29H50O2	0.75 0.30 0.43 0.42 1.18 0.85 8.93 54.94 13.20	1.30 0.57 0.86 0.32 1.77 1.13 9.33 53.50 11.74	338 268 226 422 280 402 416 416 430

2	3 5 6 10 13 15 16 17 18 19 23 26 27	7.555 10.002 12.336 14.946 17.584 18.395 18.622 18.795. 18.949 20.285 21.017 23.734 27.430	Pentadecane Hexadecane Hexadecane Heptadecan Hexadecanamide beta.-sitosterol beta-sitosterol gamma-sitosterol gamma-sitosterol 9-Octadecenamide, (Z)- Phenol,2,2′methylenebis[6-(1,1-dimethylethyl Cholest-22-ene-21-ol, 3,5-dehydro-6-methoxy-, 9,19-Cyclolanostan-3-ol, 24-methylene-, (3.beta.)-	C15H32 C16H34 C16H34 C17H36 C16H33NO C29H50O C29H50O C29H50O C29H50O C18H35NO C23H32O2 C33H54O3 C31H52O	1.59 1.48 0.88 0.47 2.54 1.09 13.47 16.41 14.46 11.27 1.71 1.67 8.30	6.21 5.45 2.99 1.35 5.54 1.13 8.16 9.22 5.67 18.32 3.33 0.68 3.58	212 226 226 240 255 414 414 414 414 281 340 498 440

18	7 8 12	30.400 30.726 32.365	beta.-Tocopherol gamma.-Tocopherol alpha.-Tocopherol-.beta.-D-mannoside	C28H48O2 C28H48O2 C35H60O7	4.24 52.30 12.59	5.83 39.73 9.61	416 416 592

20	2 3 5 6 7 10 11	26.620 26.665 30.445 30.590 30.749 31.945 32.365	Cyclotrisiloxane, hexamethyl- Periplocymarin beta-Tocopherol Heptadecafluorononanoic acid, hexyl ester gamma-Tocopherol 4-Methoxy-2(1H)-quinolone alpha.-Tocopherol-.beta.-D-mannoside	C6H18O3Si3 C30H46O8 C28H48O2 C15H13F17O2 C28H48O2 C10H9NO2 C35H60O7	2.25 3.88 3.19 2.43 51.78 4.31 3.05	6.09 2.96 4.67 3.28 36.74 5.24 7.53	222 534 416 548 416 175 592

**Table 4:** GCMS profiling of methanolic extracts of *P*. *ostreatus* mushroom grown from sugarcane bagasse substrates supplemented with varying levels of wheat bran.

Supplement (%)	Peak	RT (min)	Compound	Molecular formula	Area %	Height %	Mol wt
0	2 3 5 6 7	27.675 28.701 30.770 30.885 32.372	1,1,3,3-Tetraallyl-1,3-disilacyclobutane Octadecanoic acid, 7-hydroxy-, methyl ester gamma.-Tocopherol Ginsenol alpha.-Tocopherol-.beta.-D-mannoside	C14H24Si2 C19H38O3 C28H48O2 C15H26O C35H60O7	7.30 8.57 49.87 6.94 5.99	5.36 6.90 44.31 14.33 8.90	248 314 416 222 592

2	1 3 6	26.558 30.790 32.395	Silane, dimethyl(docosyloxy)butoxy- beta.-Tocopherol alpha.-Tocopherol-.beta.-D-mannoside	C28H60O2Si C28H48O2 C35H60O7	5.99 17.97 3.05	6.95 22.96 6.22	456 416 592

14	3 4 8 14	7.545 9.989 14.920 20.974	Pentadecane Hexadecane Heptadecane Phenol, 2,2′-methylenebis[6-(1,1-dimethylethyl)	C15H32 C16H34 C17H36 C23H32O2	2.12 2.04 0.64 0.46	3.93 3.54 0.93 0.63	212 226 240 340

18	1 4 5 7 9 11 13	5.091 7.050 7.554 9.999 11.150 12.332 13.595	Undecane 1,3-Dioxolane, 4-[[(2-methoxy-4-octadecenyl)oxy]methyl]-2,2-dimethyl- Pentadecane Hexadecane Heptadecane Hexadecane Heptadecane	C11H24 C25H48O4 C15H32 C16H34 C17H36 C16H34 C17H36	0.39 0.66 0.94 1.63 0.41 0.77 0.50	0.56 0.68 1.81 2.36 0.79 1.28 0.39	156 412 212 226 240 226 240

20	3	30.806	gamma-Tocopherol	C28H48O2	37.39	38.33	416

## Data Availability

The supplementary data used to support the results of the research study can be obtained through corresponding author upon request.
